# Prediction of the binding interactions between rosmarinic acid and cysteinyl leukotriene receptor type 1 by molecular docking and immobilized receptor chromatography

**DOI:** 10.1039/d4ra01858c

**Published:** 2024-08-01

**Authors:** Bowen Shi, Jing Wang

**Affiliations:** a Xi'an International Medical Center Hospital China; b Key Laboratory of Resource Biology and Biotechnology in Western China, Ministry of Education, College of Life Sciences, Northwest University China

## Abstract

Drug–protein interaction analysis is still at the center of research efforts to illustrate binding mechanisms and provide valuable information for selecting drug candidates with ideal properties in the early drug discovery stage. We present the prediction of the binding of rosmarinic acid (RA) to cysteinyl leukotriene receptor type1 (CysLTR1) by molecular docking. According to our findings, CysLTR1 is a potential anti-inflammatory target of RA. Under this assumption, we prepared the immobilized CysLTR1 column *via* a one-step method and characterized the immobilized CysLTR1 by fluorescent and chromatographic analyses. Furthermore, we used the immobilized CysLTR1 column to evaluate the binding interactions between RA and the immobilized receptor. Molecular docking showed that Tyr 249, Phe 174, Thr 280, Pro 177, and Thr 100 are the main sites for RA to interact with CysLTR1. The main forces that drive the findings are hydrogen bonds and hydrophobic interactions. Characterization results show that CysLTR1 is successfully immobilized with high specificity and stability. Almost no non-specific binding is observed on the immobilized CysLTR1 gels. The association constant and the binding sites are calculated to be 7.268 × 10^5^ L mol^−1^ and 1.237 × 10^−8^ mol L^−1^ by injection amount-dependent method. These results, taken together, confirm the potential target of RA on the anti-inflammatory effect. We believe that it can provide valuable reference information on the in-depth exploration of drug–protein interaction mechanisms, and lead compound screening by this method.

## Abstract 2

Drug–protein interaction analysis is significant for illustrating the binding mechanisms and providing valuable information for selecting drug candidates with ideal properties.

## Graphical abstract

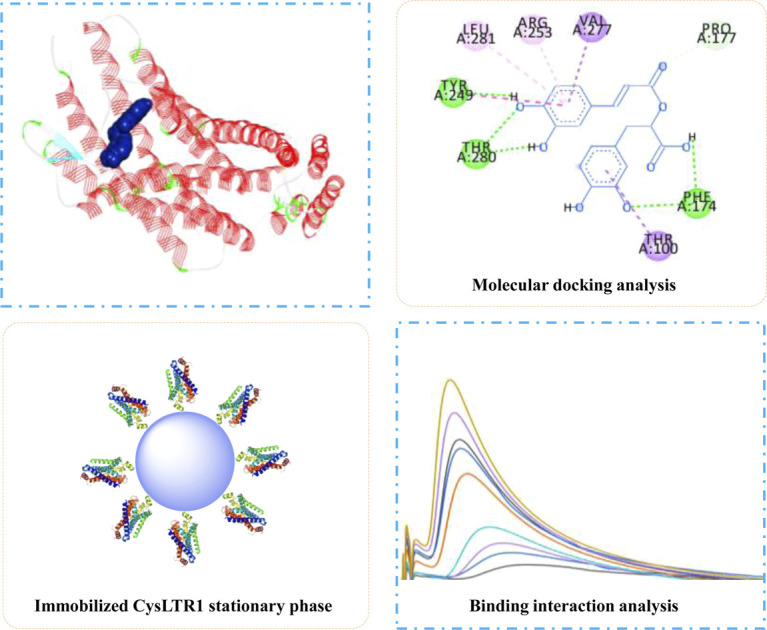

## Introduction

Drug–protein interactions and protein–protein interactions have played pivotal roles in understanding protein functions, exploring protein mechanisms, developing new therapeutic molecules, and drug discovery.^1–4^ Among these, the binding affinity investigations between the drugs and proteins have become the most interesting subject because of their crucial role in determining the pharmacological activity of the ligand.^5–9^ Thus, increased attention has been drawn to the essential role of binding affinity investigations between drugs and proteins in discovering lead compounds.

During the past few years, dramatic changes have taken place in evaluating drug–protein interactions due to the rapid development of detection technology, biotechnology, as well as instrumental technology. Commonly used methods include UV-vis absorption,^10–12^ fluorescence,^13–15^ circular dichroism,^16–18^ isothermal titration calorimetry,^19,20^ and differential scanning calorimetry.^21–23^ These methods are simple to operate and do not require the separation of proteins and drugs, however, they have high requirements for the sample purity. As an alternative, capillary electrophoresis has contributed a lot to exploring the binding interactions with low consumption of samples. Notably, the calculation of mathematical formulas needs more attention because it always varies when obtaining binding parameters. Moreover, ongoing work is needed to break their limitations in long analysis time, lacking sufficient sensitivity, relatively poor selectivity, and stability.

Chromatographic methods, especially receptor chromatography, have been popularized in revealing the binding interactions between drugs and receptors.^24,25^ It has enabled fast analysis and specific recognition into a single analysis of the analyte, which merits the method's high speed, specificity, stability, and throughput. Thus, it is superior to the conventional methods in fields, such as obtaining drug–protein interaction parameters with high precision and reproducibility. For such purposes, frontal analysis and zonal elution were mainly used to calculate the binding parameters. However, both two methods suffer from the deficiencies of long analytical periods and a large amount of sample consumption. To overcome the shortcomings of the two methods, Zhao *et al.* developed an injection-amount dependent method and non-linear chromatography for fast analysis of the binding interactions between drugs and the immobilized receptors.^24,26^ By using these methods, the binding interactions between the specific ligands and the immobilized β_2_-adrenoceptor, β_1_-adrenoceptor, muscarinic-3 acetylcholine receptor, *etc.* were investigated.^26–28^ The results showed high consistency with other classical methods in the literature.

Rosmarinic acid (RA), a water-soluble phenolic compound, has been discovered in species of the Boraginaceae family and the Nepetoideae subfamily of the Lamiaceae family. It is usually used as a defense compound for curing inflammatory diseases.^29–31^ This implies that RA has the potential to be a specific ligand of cysteinyl leukotriene receptor type 1 (CysLTR1). Herein, this work aimed to simulate the binding interaction between RA and CysLTR1 by molecular docking. Ongoing work was performed to validate the application of the immobilized CysLTR1 column in exploring the binding parameters by the injection-amount-dependent method. We believe that this strategy is promising for predictions and investigations of the bindings for other functional proteins and drugs.

## Experimental

### Materials and instruments

Standards of pranlukast (Cat. No. S60198), zafirlukast (Cat. No. S80074), MK-571 (Cat. S80126), and rosmarinic acid (Cat. No. B20862) with purities greater than 98% were purchased from Shanghai Yuanye Bio-Technology Co., Ltd. A pre-stained protein marker (Cat. No. G2058) was bought from Wuhan Service Bio Technology Co., Ltd. Tryptone (Cat. No. LP0042) and yeast powder (Cat. No. LP0021) were purchased from Oxoid Company, UK. Macro-porous silica gel with a particle size of 7.0 μm and a core size of 300 Å was bought from Suzhou Knowledge & Benefit Sphere Tech. Co., Ltd.

The centrifuge, with a Cat. No. of Sorvall LYNX 4000, was used to separate the supernatant. The precipitant of the cell lysate was obtained from ThermoFisher Scientific (China) Co., Ltd. The ultrasonic cell crusher (Cat. No. JY92-IIN) was bought from Ningbo Xinzhi Biotechnology Co., Ltd, which was used to disrupt the cells that produced the target proteins. Furthermore, we used a ZZXT-A packing machine purchased from Dalian Elite Analytical Instrument Co., Ltd to prepare the immobilized CysLTR1 column. Chromatographic experiments were performed by the Shimadzu LC-2030 high-performance liquid chromatography apparatus with an isocratic pump, an autosampler, a column temperature chamber, and a UV detector (Shimadzu Inc., Japan).

### Molecular simulations

The crystal structure of CysLTR1 was downloaded from the Protein Data Bank (PDB ID: 6RZ5). We kept the receptor in rigid mode for the simulation process. The pre-bonded small ligands, such as zafirlukast, water, and ions, were removed by PyMOL. The crystal structure was pre-treated by adding polar hydrogens and Gasteiger charges before docking. For the three drugs and the screened bioactive compound, the structures were generated by ChemDraw Ultra 8.0. A rectangular box enclosing the ligand-binding site of zafirlukast in CysLTR1 was applied as the sampling space for docking. The size for the grid box was set as 60 Å × 60 Å × 60 Å. We performed three independent docking runs and selected the binding pose with the lowest docking score. We analyzed and visualized the receptor–ligand complex using Discovery Studio 4.5.

### Expression of Halo-tagged CysLTR1

We transformed the fused Halo-tagged CysLTR1 plasmid into *Escherichia coli* (*E. coli*) BL21(DE3) cells by the heat-shock method. The cells were then spread onto a 25 mL Luria-Bertani (LB) agar plate and incubated overnight at 37 °C with ampicillin at a final concentration of 100 μg mL^−1^. The next day, we picked up a single colony and transferred it into 150 mL of LB medium for another round of growth at 37 °C under an ampicillin atmosphere. After that, the cells were transferred into an auto-induction medium for expression of the target protein at 37 °C for 10 hours. Finally, we harvested 8.5 g of the cells by centrifugation at 4 °C for 20 min with a rotating speed of 7000 rpm. The harvested cells were then re-suspended in 20 mM phosphate buffer (PB, pH = 7.40), disrupted, and centrifuged at 4 °C for 30 min. All the samples were then collected for further use.

### Immobilization of CysLTR1

Using a conventional construction process from the literature,^32^ we prepared the stationary phase by the following procedures: (1) activation of the silica gel. Briefly, 1.0 g of dried aminopropyl silica gel was weighed and transferred into a flask. The gels were then suspended in 10.0 mL of dimethylformamide (DMF) within 82.98 mg of 6-chlorohexanoic acid (1.2 eq.) and 240 μL of *N*,*N*-diisopropyl ethylamine (3.0 eq.). Sequentially, 210 mg of 2-(7-azabenzotriazol-1-yl)-*N*,*N*,*N*′,*N*′-tetramethyluronium hexafluorophosphate was added into the flask, and the reaction was allowed to occur for 2.0 hours at ambient temperature. After that, the gel was filtered and sequentially rinsed with DMF and 20 mM phosphate buffer (PB, pH = 7.40). (2) Immobilization of CysLTR1. The gels were re-suspended into 50 mL of the supernatant of the cell lysate containing the expressed CysLTR1. The gels were then reacted for 1.0 h to prepare the immobilized receptor. The immobilized CysLTR1 was then packed into the stainless-steel column (4.6 × 30 mm) using 20 mM PB (pH = 7.40) as slurry and propulsive agent under the pressure of 400 bar for 1 hour.

### Characterization of the immobilized CysLTR1

#### Fluorescent analysis

We prepared the bare silica gel, amino-functionalized gel, 6-chlorohexanoic acid modified gel, and CysLTR1 coated gel through the aforementioned procedures. 200 μg of these gels were incubated with 1 μL of cyanine5 maleimide for 1.0 h. The gels were then washed with 20 mM PB (5.0 mL × 3) and examined under the fluorescence microscope with an excitation wavelength of 670 nm.

#### Chromatographic analysis

Twenty millimolar PB (pH = 7.40) was taken as the mobile phase. We injected four drugs into the immobilized CysLTR1 column to test their binding behaviors on the immobilized CysLTR1 column. The injection volume and the flow rate were set to 10 μL and 0.2 mL min^−1^. The detection wavelengths were 262 nm for pranlukast, 242 nm for zafirlukast, 238 nm for MK-571, and 205 nm for sodium nitrite, respectively. Isocratic elution mode was performed during the immobilized CysLTR1 chromatography with 20 mM PB (pH = 7.40) as the elution buffer throughout the elution process. The flow rate was set the same as before. Taking zafirlukast as the probe, we tested the stability of the chromatographic system for 30 days.

### Binding interaction investigations

We applied the injection amount-dependent method and nonlinear chromatography to investigate the binding interactions between the drugs and the immobilized CysLTR1 at 37 °C. We prepared the stock solution in a final concentration of 5 mM for each drug. We prepared the analyte by diluting the stock solutions with 20 mM PB (pH = 7.40). The concentrations were 0.1, 0.2, 0.3, 0.4, 0.5, 0.6, 0.7, 0.8, 0.9, and 1.0 mM for zafirlukast; 0.2, 0.3, 0.4, 0.5, 0.6, 0.7, 0.8, 0.9, and 1.0 mM for pranlukast; 0.3, 0.4, 0.5, 0.6, 0.7, 0.8, 0.9, and 1.0 mM for MK-571, and 0.3, 0.4, 0.5, 0.6, 0.7, 0.8, 0.9, 1.0, and 1.1 mM for RA. After passing through the 0.45 μm filter membrane, we loaded the drugs onto the column containing the immobilized CysLTR1 in triplicates. The injection volume and the flow rate were set at 10 μL and 0.6 mL min^−1^. The wavelengths detected for the three drugs were consistent with the aforementioned ones, while for RA, it was 330 nm. The mobile phases were 50 mM PB in 2-propanol (91 : 9, V : V) for pranlukast and zafirlukast; it was 20 mM PB (pH = 7.40) for MK-571 and RA, respectively. We employed isocratic elution to investigate the binding parameters between the drugs and immobilized CysLTR1. The elution solutions and the flow rate we used here were the same as the ones mentioned before. All the chromatographic profiles were recorded, and the data were analyzed.

## Results and discussion

### Molecular docking analysis

As reported in the literature, the ligand binding pocket of CysLTR1 extends from the extracellular loop2(ECL2) across the receptor toward a gap between the transmembrane helices, 4 (TM4) and 5 (TM5), then deep into the middle part of the TM 7.^33^ Tyr 104 and Tyr 249 residues that are located at the ECL2 sections are demonstrated to create polar interactions with the ligand. Arg 79 on the side chain is proven to participate in the formation of salt bridges and is involved in hydrophobic interactions with the ligands. Furthermore, some amino acids in the CysLTR1, *i.e.*, Phe 158, Tyr 108, Ser 155, Leu 189, His 190, Arg 253, His 256, Pro 176, Val 277, Thr 100, and Leu 281, were involved in the binding pockets and were identified as major sites for the binding of ligands by hydrogen bonds and hydrophobic interactions.

**Fig. 1:**
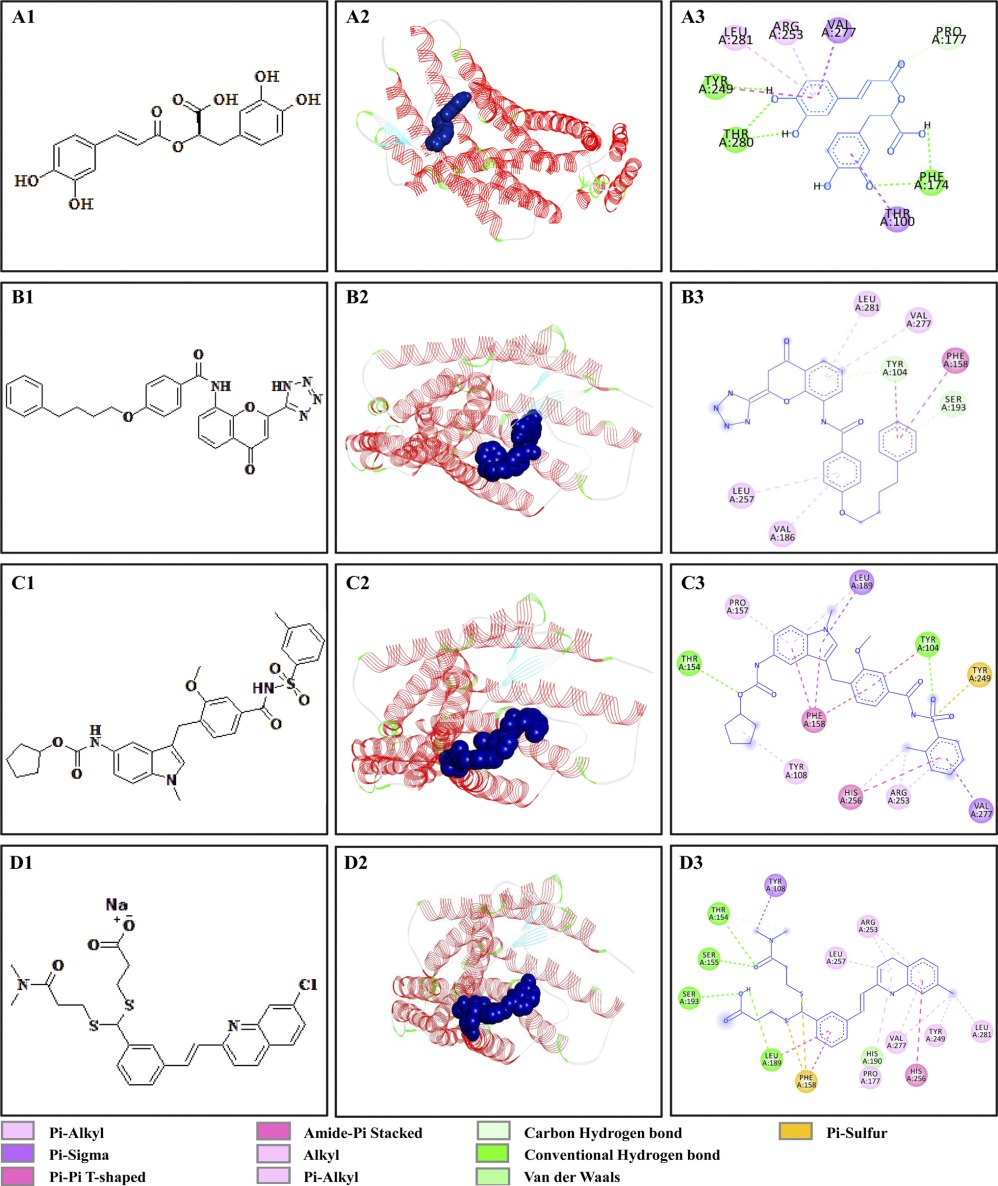
Molecular docking analysis of the binding interaction between the four drugs and CysLTR1. (A1–D1), chemical structures of rosmarinic acid, pranlukast, zafirlukast and MK-571. (A2–D2) and (A3–D3) were 3D and 2D overviews of the binding interactions between the four drugs and CysLTR1 by molecular docking. The receptor was shown as ribbon and the drugs were displayed as spheres.

The chemical structures, 3D and 2D overviews of the molecular simulations, between RA, pranlukast, zafirlukast, MK-571, and CysLTR1 are displayed in Fig. 1. The results revealed that specific interactions occurred between RA and CysLTR1. The amino residues, Phe 174, Thr 280, Pro177, and Tyr 249 on CysLTR1 participated in the formation of hydrogen bonds with RA. While Leu 281, Arg 253, Val 277, and Thr 100, mainly interacted with RA by hydrophobic interactions through the benzene ring of RA. Meanwhile, we found that hydrogen bonds, van der Waals force, and hydrophobic interactions were the main driving forces for pranlukast, zafirlukast, and MK-571 to bind to CysLTR1. The residues were located all around the binding pocket of the receptor. Taken together, these results demonstrated that the specific binding sites of RA to CysLTR1 were consistent with the results from the literature, reminding us that RA is a potential anti-inflammatory ligand of CysLTR1.

### Expression of Halo-tagged CysLTR1

As a fundamental step, the expression of the target proteins plays a pivotal role in the preparation of functional protein columns for subsequent investigations, especially regarding the largest *trans*-membrane protein family members: G protein-coupled receptors. In this work, we expressed the Halo-tagged CysLTR1 by a previously reported method.^34^ A prokaryotic system, *Escherichia coli* (*E. coli*) cell, was used to express the target protein because it benefits the method of low cost and fast growth rate. Briefly, a heat-shock method was used to transform the fused Halo-tagged CysLTR1 plasmid into the *E. coli* BL21 (DE3) cells. According to the conventional target protein expression manual, the Halo-tagged CysLTR1 was induced in an LB medium at different temperatures using varied concentrations of isopropyl-beta-d-thiogalactopyranoside (IPTG) as the inducer. However, the outcome was not satisfying because the target protein cannot be expressed very well by using such a method. We attributed this result to the low stability and the aggregation of the recombinant protein by using the methods mentioned above. As an alternative, the auto-induction medium was applied to replace the conventional IPTG-based induction due to its simple and soluble expression.^35,36^ When the target recombinant protein is in a soluble and biologically active form, the inefficient refolding of the protein can be ignored, and thus, it has been proven to be advantageous to use the target protein as a template.

**Fig. 2:**
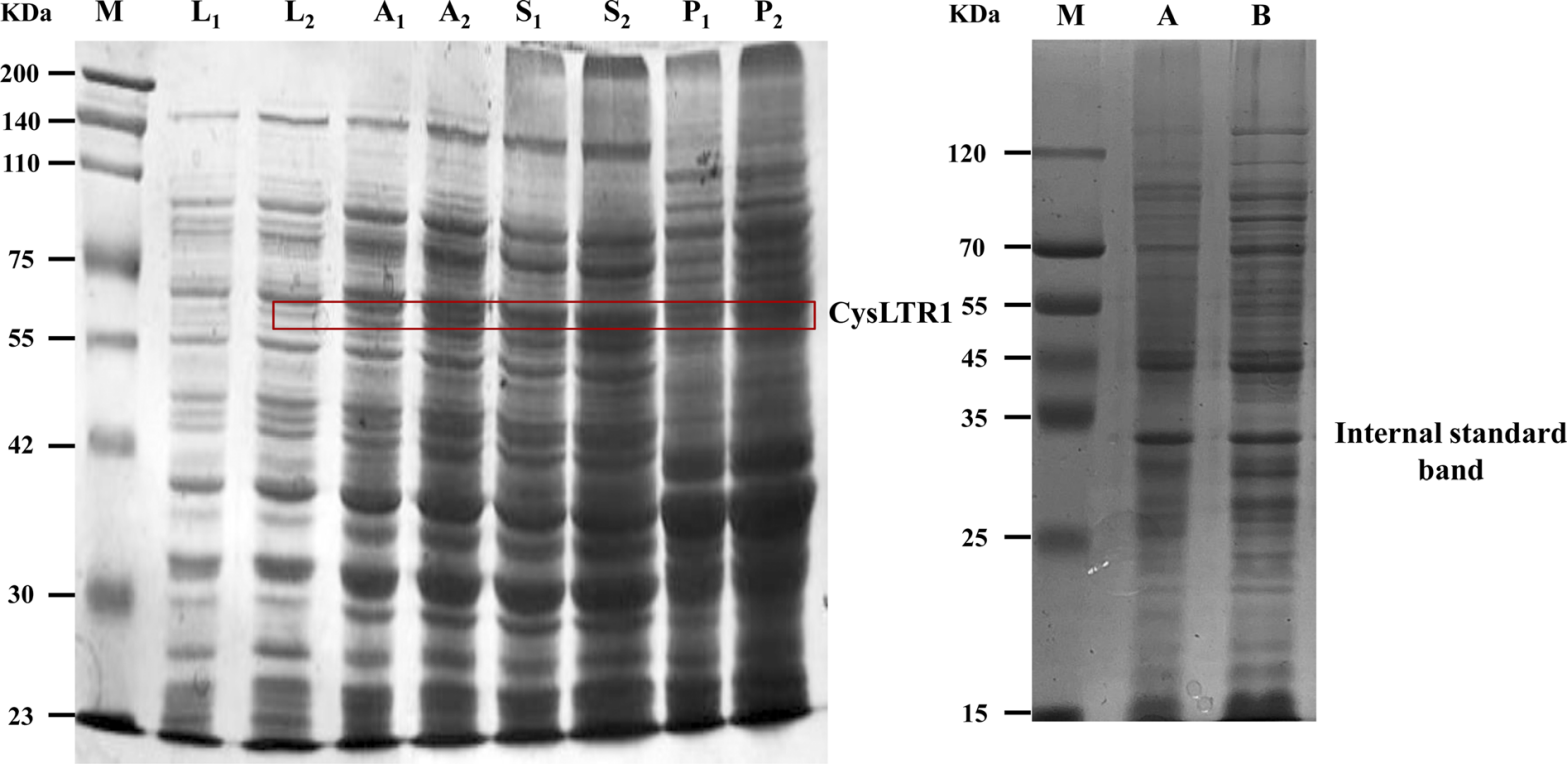
SDS-PAGE analysis of CysLTR1. Lane M: protein marker, lane L_1_, L_2_: LB medium, lane A_1_, A_2_: auto-induction medium, lane S_1_, S_2_: supernatant of the cell lysate, lane P_1_, P_2_: precipitant of the cell lysate. Lane A: supernatant of the cell lysate after immobilization, lane B: supernatant of the cell lysate before immobilization. The subscript 1 and subscript 2 referred to the loading volume of the analyte as 10 μL and 20 μL.

Fig. 2 shows sodium dodecyl sulfate-polyacrylamide gel electrophoresis (SDS-PAGE) analysis of the expressed Halo-tagged CysLTR1. We found that a new band appeared in the auto-induction medium located at the position corresponding to the molecular weight between 55 kDa and 75 kDa. By calculating the molecular weight of the newly appeared band, we determined it to be Halo-tagged CysLTR1 as the value highly agreed well with the theoretical molecular weight of the protein. From this point of view, we believe that the target protein has been successfully expressed. Meanwhile, we found that in comparison with the supernatant, this new band was rarely expressed in the precipitant. Even if the loading volume increased to 20 μL, there were no significant changes. We considered that this outcome is rational because the target protein is mainly expressed in soluble forms in the supernatant, therefore, it can retain its bioactivity. Thus, the content of the target band in the precipitant is very low. In brief, these findings indicate that the recombinant protein is successfully expressed in the supernatant of the cell lysate.

**Fig. 3:**
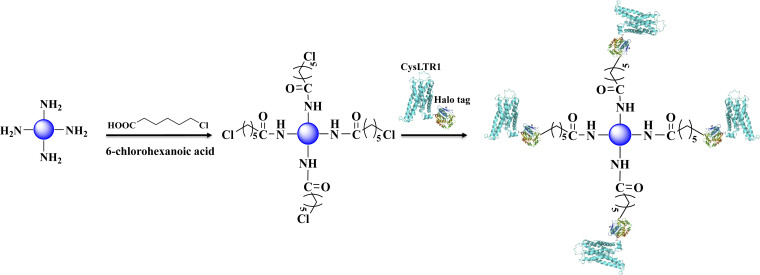
Immobilization of CysLTR1 *via* one-step relying on the specific interaction between haloalkane dehalogenase and 6-chlorohexanoic acid.

In this work, we immobilized CysLTR1 through the specific interaction between haloalkane dehalogenase and 6-chlorohexanoic acid (Fig. 3). The content of the target protein was determined by analyzing the difference in grayscale of the target protein band before- and after-immobilization by SDS-PAGE (Fig. 1). The content of the immobilized receptor percentage was calculated to be 3.86% (before) and 1.42% (after) by using a band as the internal standard. In this way, the amount of the immobilized CysLTR1 was calculated to be 18.42 mg g^−1^. Furthermore, we used fluorescent analysis and chromatographic investigations to test whether CysLTR1 was successfully immobilized onto the gel surface.

#### Fluorescent analysis

Fluorescent analysis is a frequently used method to detect biomolecules due to their high sensitivity, good reproducibility, high specificity, and ease of use.^37,38^ It usually employs fluorophore-labeled materials, or specific antibodies to detect the concentration of the target bio-molecule using the concentration of the fluorescence intensity. Benefiting from the advantages of fluorescent analysis, it has been popularized in fields, such as chemistry, materials science, physical, medical, environmental, and biotechnological applications. Some modern techniques, such as fluorescence sensors, have been developed to detect biomolecules, such as fluorescent sensors, fluorescence microscopy, and enzyme-assisted fluorescence techniques. In this set of experiments, we intend to detect the immobilized CysLTR1 based on the fluorescent method.

**Fig. 4:**
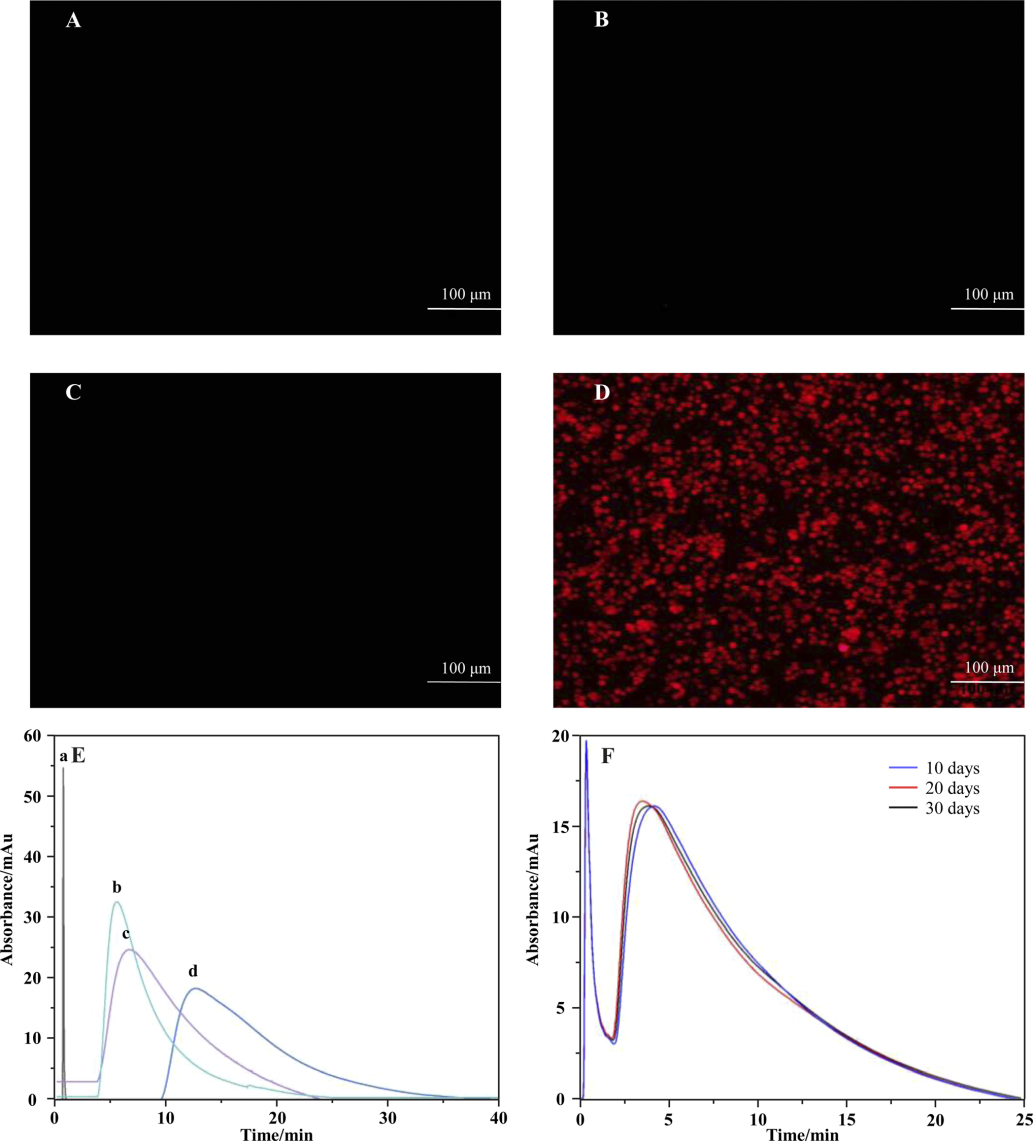
Characterization of the immobilized CysLTR1. (1) Fluorescent analysis of the bare silica gel (A), amino-functionalized gel (B), 6-chlorohexanoic acid modified gel (C), and CysLTR1 coated gel (D) with the magnification of 200×. (2) specificity characterization (E) of the immobilized CysLTR1 column. a, b, c, and d were sodium nitrite, zafirlukast, MK-571, and pranlukast on the immobilized CysLTR1 column; (F) representative chromatograms of zafirlukast on the CysLTR1 column in 30 days.

Fig. 4 displays the bare silica gel, amino-functionalized silica gel, 6-chlorohexanoic acid-modified gel, and CysLTR1-coated gel incubated with a specific substrate-cyanine 5 maleimide for 1.0 h. Cyanine 5 maleimide is a kind of dye that has been used to label biological molecules for fluorescence imaging and other fluorescence-based biochemical analyses. It is usually recommended for labeling thiol groups of the molecules. Herein, we used it to label the immobilized CysLTR1 onto the surface of the silica gel. We found that rare fluorescent signals appear in the bare silica gel, amino-functionalized silica gel, as well as 6-chlorohexanoic acid-modified gel. Compared with these samples, CysLTR1-modified silica gel showed significant fluorescent signals. The result was justified because only the structures of Halo-tagged CysLTR1 contained thiol groups, thus it can be labeled, while the other three groups could not be labeled. Thus, we believed that Halo-tagged CysLTR1 was successfully immobilized onto the gel surface.

#### Chromatographic analysis

We investigated the specificity of the immobilized CysLTR1 column by testing the capacity factors of pranlukast, zafirlukast, and MK-571 on the column with the values on the control column, which was packed with bare silica gel as the stationary phase. No substantial differences were observed in the capacity factors for these columns, suggesting that nonspecific interactions rarely occurred between the drugs and the immobilized CysLTR1. The specificity of the column containing the immobilized CysLTR1 was tested by determining the chromatographic profiles and the retention times of the three drugs on the immobilized CysLTR1 column. The representative chromatographic profiles of sodium nitrite, pranlukast, zafirlukast, and MK-571 are depicted in Fig. 4. We found that the four drugs presented different retention times on the immobilized CysLTR1 column. Meanwhile, the retention times of the three drugs, pranlukast, zafirlukast, and MK-571, were all longer than the retention time of sodium nitrite. This result is reasonable because sodium nitrite is a non-specific ligand of CysLTR1. Thus, we attributed the retention time of sodium nitrite to the void time of the chromatographic system. In addition, the other three drugs presented longer, but varied, retention times than sodium nitrite. Retention times were 0.298 min for sodium nitrite, 5.281 min for zafirlukast, 7.234 min for MK-571, and 8.358 min for pranlukast, respectively. This result is rational as the three drugs behaved as specific ligands of CysLTR1. From this aspect, we reasoned that variation in the retention times confirmed that the immobilized receptor still retained specificity for its ligands after immobilization and packing into the stainless-steel column.

Furthermore, the stability of the immobilized CysLTR1 column was tested using MK-571 as a probe. We tested it by injecting the drug into the column for 30 consecutive 30 days. The representative chromatograms of MK-571 on the immobilized CysLTR1 column are depicted in Fig. 4F. The results demonstrate that no observable difference emerged in either the chromatographic profiles or the retention times of the drug. It proved that the column containing the immobilized CysLTR1 can be stable for at least 30 days. The column could be re-used for as many as 900 injections. The relative standard deviation of the retention times for MK-571 was 0.5%. In this case, the immobilized CysLTR1 proved to be stable for at least 30 days with a sampling efficiency of 900 injections. Such results provide evidence of the stability of the column. Taken together, the immobilized CysLTR1 column was proved to be stable for at least one month.

### Binding interaction analysis

**Fig. 5:**
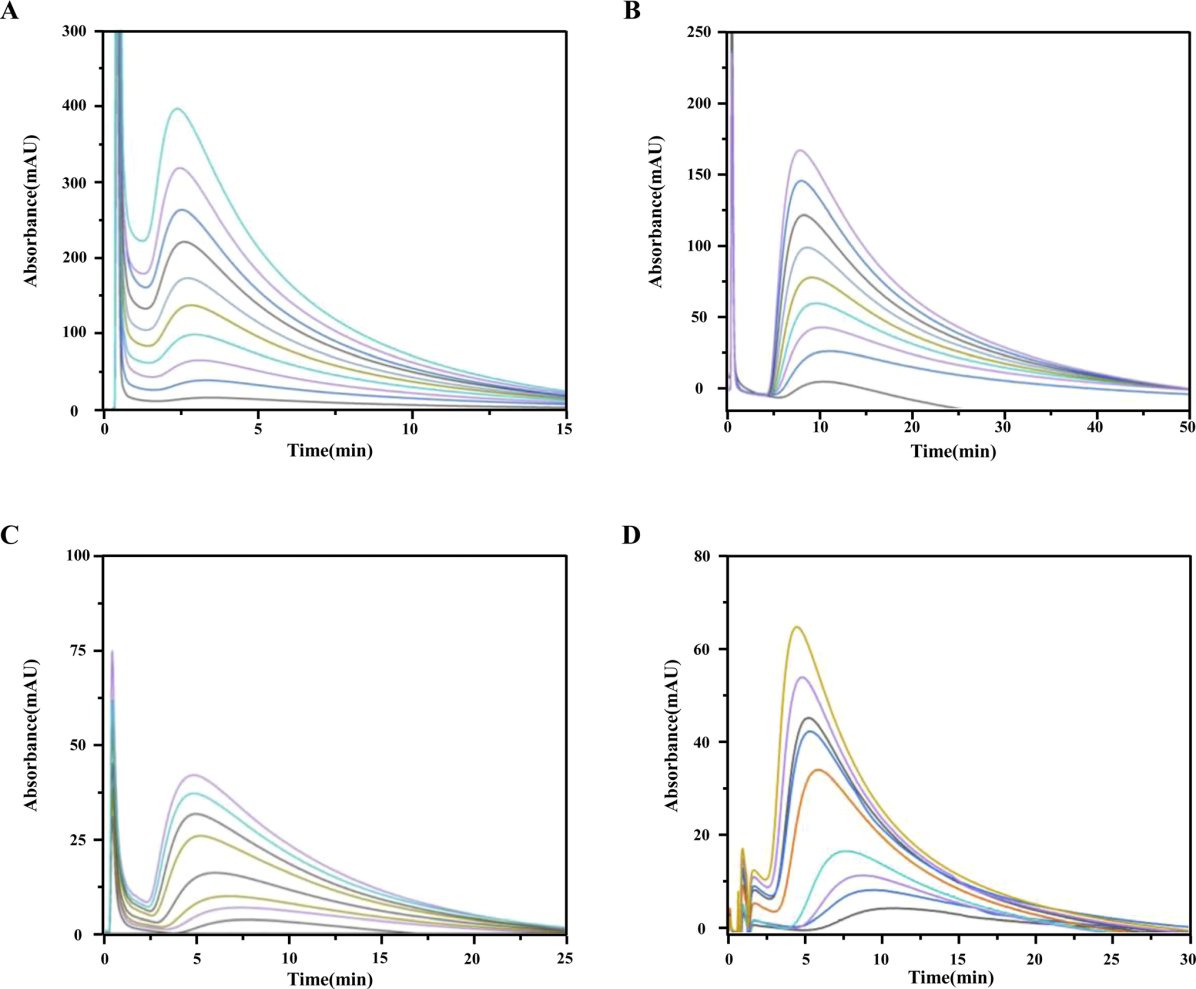
Representative chromatograms of the four ligands on the immobilized CysLTR1 column. The concentrations were 0.1, 0.2, 0.3, 0.4, 0.5, 0.6, 0.7, 0.8, 0.9 and 1.0 mm for zafirlukast (A), 0.2, 0.3, 0.4, 0.5, 0.6, 0.7, 0.8, 0.9 and 1.0 mm for pranlukast (B), 0.3, 0.4, 0.5, 0.6, 0.7, 0.8, 0.9 and 1.0 mm for MK-571 (C), 0.3, 0.4, 0.5, 0.6, 0.7, 0.8, 0.9, 1.0, and 1.1 mm for rosmarinic acid (D). The detection wavelength was 242 nm, 262 nm, 238 nm, and 330 nm for the four drugs, respectively. The flow rate and the injection volume were set as 0.6 mL min^−1^ and 10 μL.

**Fig. 6:**
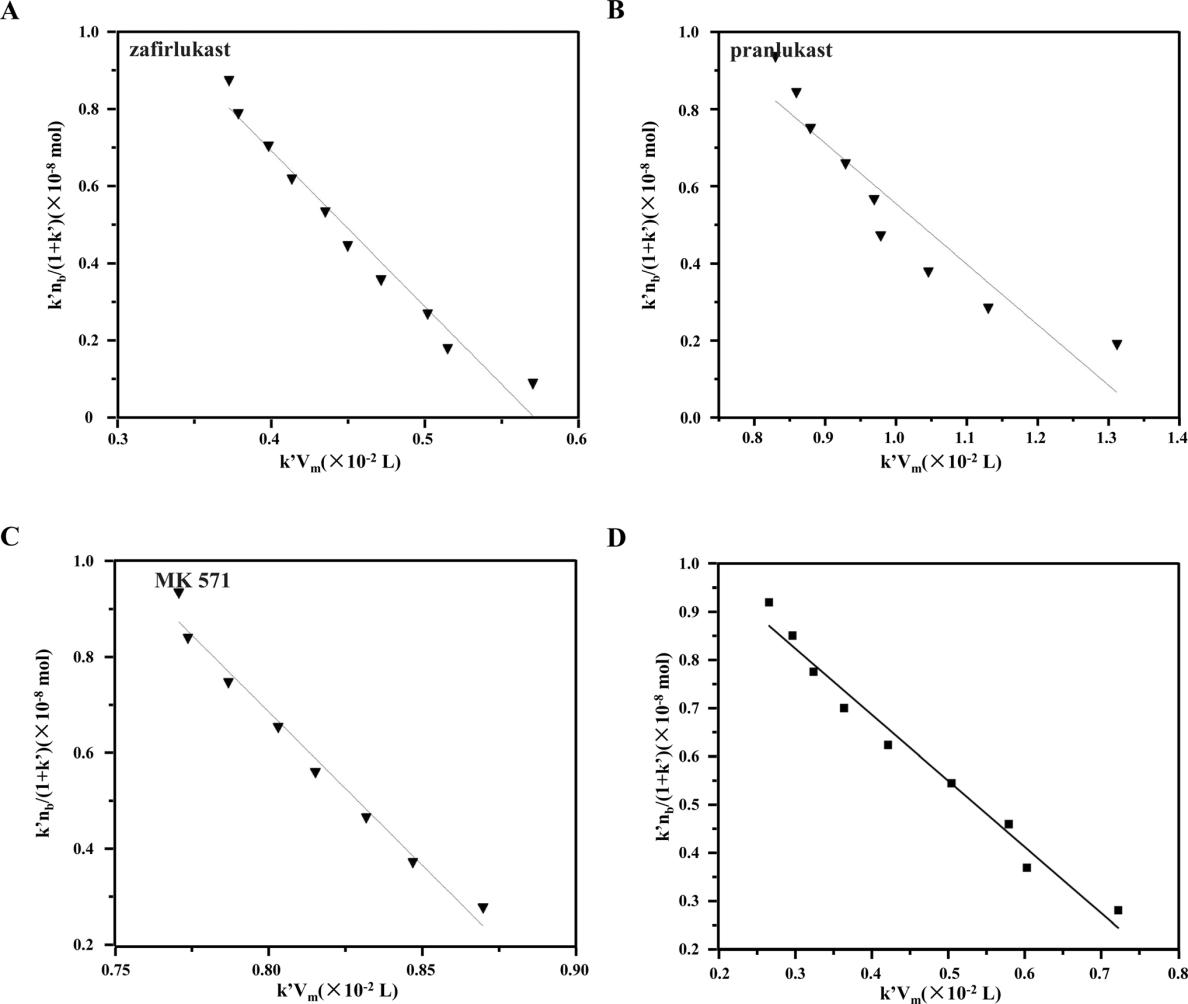
The plots of *k*′*n*_b_/(1 + *k*′) *versus k*′*V*_m_ for the zafirlukast (A), pranlukast (B), MK-571 (C), and rosmarinic acid (D) on the immobilized CysLTR1 column determined by injection of various concentration of each drug. the equations of the best-fit lines were *y* = −4.02 × 10^− 6^*x* + 2.30 × 10^−8^ for zafirlukast; *y* = −2.09 × 10^− 6^*x* + 2.6 × 10^−8^ for pranlukast; *y* = −2.43 × 10^− 6^*x* + 2.11 × 10^−8^ for MK-571, and *y* = −1.376 × 10^− 6^*x* + 1.24 × 10^−8^ for rosmarinic acid. the corresponding correlation coefficient were 0.9686, 0.9511, 0.9674, and 0.9771 for the four drugs, respectively.

The binding and elution of the drug on the immobilized receptor chromatography is similar to the absorption and desorption processes *in vivo*. Thus, we studied the immobilized CysLTR1 to investigate the binding interaction between drugs and the receptor. In this situation, isocratic elution was used to allow a single mobile phase to be used for both sample application and elution in the immobilized CysLTR1 column. The elution buffer we used was the same as the mobile phase to illustrate the binding interactions by the injection-amount-dependent method. It is believed to be a convenient and reliable method that was first proposed in 2014. Unlike frontal analysis and zonal elution,^39,40^ this method does not need to saturate the binding sites of the immobilized CysLTR1 column, thus it is time- and less labor-intensive. When using this method to explore the binding parameters of the given drug, we usually assume that the binding of the drug to the immobilized receptor occurs rapidly. The adsorption sites are homogeneously distributed on the surface of the stationary phase, while the longitudinal diffusion can be ignored. Therefore, we can obtain the association constants from eqn (1) using the relationship between the molar amount of the injected solute and its capacity factor:1
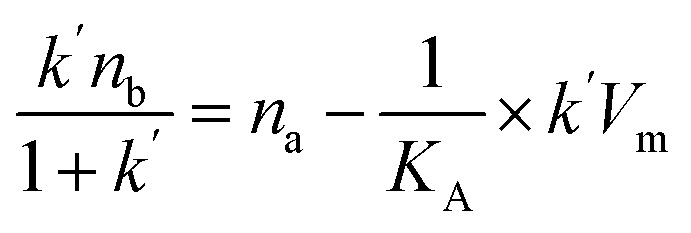
*k*′ represents the capacity factor of the tested drugs on the immobilized CysLTR1 column. *n*_b_, *K*_A_, and *V*_m_ stand for the molar amount of one injection, the association constant, and the void volume of the chromatographic system. *n*_a_ denotes the total amount of the binding sites of the drug on the column. From eqn (1), a linear regression relationship between 
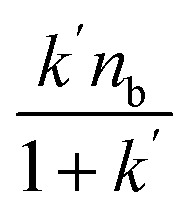
 and *k*′ appears. Thus, we can obtain the association constant and the number of binding sites through the slope and the intercept of the linear relationship. In this set of experiments, we injected a series of concentrations of drugs into the column containing immobilized CysLTR1. Representative chromatograms of the four drugs with different concentrations of each drug injected into the immobilized CysLTR1 column are presented in Fig. 5 under the proposed chromatographic conditions. We found that though the concentration of each drug on the immobilized CysLTR1 column varied, the chromatograms were all similar with a tailing peak. Meanwhile, the retention time of each drug decreased when the injection concentration of the drug increased. The variations in the capacity factors of each drug negatively corresponded to the increased concentration of the drug. This phenomenon is reasonable as the active binding sites of the immobilized CysLTR1 in the column are constant. With the increasing concentration of the injected drugs, a decreased retention time was observed on the column. This result agrees well with the assumption of the injection amount-dependent method. Thus, using eqn (1), the linear relationships between *k*′*n*_b_/(1 + *k*′) *versus k*′*V*_m_ for the four drugs are depicted in Fig. 6. Based on the equations, the association constant, and the number of binding sites are calculated to be 2.193 × 10^5^ L mol^−1^, 2.299 × 10^−8^ mol L^−1^ for zafirlukast, 4.789 × 10^5^ L mol^−1^, 2.598 × 10^−8^ mol L^−1^ for pranlukast, 4.272 × 10^5^ L mol^−1^, 2.094 × 10^−8^ mol L^−1^ for MK-571, and 7.268 × 10^5^ L mol^−1^, 1.237 × 10^−8^ mol L^−1^ for RA, respectively. The order in which the association constants of the three ligands could be ranked for the immobilized CysLTR1 is pranlukast > MK-571 > zafirlukast, which agrees well with the references. Thus, we believe that the established immobilized CysLTR1 chromatography can be used for exploring the binding parameters of drugs.

## Conclusions

Herein, in this work, we predicted and investigated the potential binding of rosmarinic acid to CysLTR1 using molecular docking and injection amount-dependent methods. It was found that Phe 158, Tyr 108, Ser 155, Leu 189, His 190, Arg 253, His 256, Pro 176, Val 277, Thr 100, and Leu 281 on CysLTR1 were the main amino acid residues that participated in the binding of drugs to the receptor. The feasibility of the immobilized CysLTR1 column was investigated by injection-amount dependent method to explore the binding parameters of the four drugs. The results reveal that it has the potential to become a powerful tool for the design and screening of important compounds that specifically bind to the target protein by immobilized receptor chromatography.

## Data availability

The data that support the findings of this study are available from the corresponding author upon reasonable request.

## Author contributions

Bowen Shi: methodology, investigation, data curation, validation, writing original draft. Jing Wang: conceptualization, methodology, validation, formal analysis, supervision, funding acquisition.

## Conflicts of interest

There are no conflicts to declare.

## References

[cit1] Wang L. J., Zhang W. M., Shao Y. L., Zhang D. T., Guo G. S., Wang X. Y. (2022). Anal. Chim. Acta.

[cit2] Wanat K., Brzezinska E., Sobanska A. W. (2018). Curr. Pharmaceut. Des..

[cit3] Seyfinejad B., Ozkan S. A., Jouyban A. (2021). Talanta.

[cit4] Liu X., Fang M., Xu F., Chen D. (2019). Trends Anal. Chem..

[cit5] Premetis G. E., Labrou N. E. (2020). Biomolecules.

[cit6] Lecas L., Dugas V., Demesmay C. (2021). Separ. Purif. Rev..

[cit7] Rodriguez E. L., Poddar S., Iftekhar S., Suh K., Woolfork A. G., Ovbude S., Pekarek A., Walters M., Lott S., Hage D. S. (2020). J. Chromatogr. B.

[cit8] Zheng X., Matsuda R., Hage D. S. (2014). J. Chromatogr. A.

[cit9] Anguizola J., Joseph K. S., Barnaby O. S., Matsuda R., Alvarado G., Clarke W., Cerny R. L., Hage D. S. (2013). Anal. Chem..

[cit10] Almutairi F. M., Ajmal M. R., Siddiqi M. K., Amir M., Khan R. H. (2020). J. Mol. Struct..

[cit11] Benesi H. A., Hildebrand J. H. (1949). J. Am. Chem. Soc..

[cit12] Yasmeen S., Riyazuddeen, Khatun S., Abul Qais F. (2020). J. Biomol. Struct. Dyn..

[cit13] Yong L., Huang M., Wei Y., Xu J., Yi Z. (2020). Anal. Methods.

[cit14] Makarska-Bialokoz M. (2018). Spectrochim. Acta Mol. Biomol. Spectrosc..

[cit15] Ciotta E., Prosposito P., Pizzoferrato R. (2019). J. Lumin..

[cit16] Greenfield N. J. (2006). Nat. Protoc..

[cit17] Collin F., Cerlati O., Couderc F., Lonetti B., Marty J. D., Mingotaud A. F. (2020). Trends Anal. Chem..

[cit18] Francis J. A., Shalauddin M., Ridzwan N. F. W., Mohamad S. B., Basirun W. J., Tayyab S. (2020). Spectrosc. Lett..

[cit19] Baranauskiene L., Kuo T.-C., Chen W.-Y., Matulis D. (2019). Curr. Opin. Biotechnol..

[cit20] Daems E., Moro G., Campos R., De Wael K. (2021). Trends Anal. Chem..

[cit21] Wang S., Yu S., Siedler M., Ihnat P. M., Filoti D. I., Lu M., Zuo L. (2018). Sensor. Actuator. B Chem..

[cit22] Berrío Escobar J. F., Márquez Fernández D. M., Giordani C., Castelli F., Sarpietro M. G. (2019). J. Pharm. Pharmacol..

[cit23] Amani M., Moosavi-Movahedi A. A., Kurganov B. I. (2017). Int. J. Biol. Macromol..

[cit24] Zhao X. F., Li Q., Chen J. J., Xiao C. N., Bian L. J., Zheng J. B., Zheng X. H., Li Z. J., Zhang Y. Y. (2014). J. Chromatogr. A.

[cit25] Jia X. N., Liu J. J., Shi B. M., Liang Q., Gao J., Feng G. J., Chang Z. M., Li Q., Zhang X. H., Chen J. B., Zhao X. F. (2019). Front. Pharmacol.

[cit26] Zhao X., Fu X. Y., Yuan X. Y., Shayiranbieke A. E. D. S., Xu R., Cao F., Ren J. P., Liang Q., Zhao X. F. (2021). J. Chromatogr. A.

[cit27] Shayiranbieke A. E. D. S., Liang Q., Wang T. T., Ma J., Li G. A., Du X. Q., Zhang G. D., Wang C. Z., Zhao X. F. (2022). J. Chromatogr. A.

[cit28] Liu G. X., Wang P., Li C., Wang J., Sun Z. Y., Zhao X. F., Zheng X. H. (2017). J. Mol. Recognit..

[cit29] Luo W. B., Tao Y., Chen S. N., Luo H., Li X. P., Qu S., Chen K., Zeng C. Y. (2022). Front. Pharmacol.

[cit30] Luo C. X., Zou L., Sun H. J., Peng J. Y., Gao C., Bao L. C., Ji R. P., Jin Y., Sun S. Y. (2020). Front. Pharmacol.

[cit31] Colica C., Di Renzo L., Aiello V., De Lorenzo A., Abenavoli L. (2018). Rev. Recent Clin. Trials.

[cit32] Zeng K. Z., Li Q., Wang J., Yin G. W., Zhang Y. J., Xiao C. N., Fan T. P., Zhao X. F., Zheng X. H. (2018). Chem. Sci..

[cit33] Luginina A., Gusach A., Marin E., Mishin A., Brouillette R., Popov P., Shiriaeva A., Besserer-Offroy É., Longpré J. M., Lyapina E., Ishchenko A., Patel N., Polovinkin V., Safronova N., Bogorodskiy A., Edelweiss E., Hu H., Weierstall U., Liu W., Batyuk A., Gordeliy V., Han G. W., Sarret P., Katritch V., Borshchevskiy V., Cherezov V. (2019). Sci. Adv..

[cit34] Feng G. J., Yuan X. Y., Li P., Tian R., Hou Z. L., Fu X. Y., Chang Z. M., Li Q., Zhao X. F. (2021). J. Chromatogr. A.

[cit35] Fathi-Roudsaria M., Maghsoudib N., Maghsoudic A., Niazia S., Soleimana M. (2018). Protein Expr. Purif..

[cit36] William Studier F. (2005). Protein Expr. Purif..

[cit37] Hu X. Y., Tan W. Q., Cheng S. S., Xian Y. Z., Zhang C. L. (2023). Anal. Bioanal. Chem..

[cit38] Chen G. Y., Chai T. Q., Wang J. L., Yang F. Q. (2023). J. Pharmaceut. Biomed..

[cit39] Hage D. S. (2002). J. Chromatogr. B.

[cit40] Kasai K. (2020). Methods Mol. Biol..

